# The 2023 Impact of Inflammatory Bowel Disease in Canada: Cancer and IBD

**DOI:** 10.1093/jcag/gwad006

**Published:** 2023-09-05

**Authors:** Sanjay K Murthy, M Ellen Kuenzig, Joseph W Windsor, Priscilla Matthews, Parul Tandon, Eric I Benchimol, Charles N Bernstein, Alain Bitton, Stephanie Coward, Jennifer L Jones, Gilaad G Kaplan, Kate Lee, Laura E Targownik, Juan-Nicolás Peña-Sánchez, Noelle Rohatinsky, Sara Ghandeharian, Saketh Meka, Roxana S Chis, Sarang Gupta, Eric Cheah, Tal Davis, Jake Weinstein, James H B Im, Quinn Goddard, Julia Gorospe, Jennifer Loschiavo, Kaitlyn McQuaid, Joseph D’Addario, Ken Silver, Robyn Oppenheim, Harminder Singh

**Affiliations:** Department of Medicine, University of Ottawa, Ottawa, Ontario, Canada; The Ottawa Hospital IBD Centre, Ottawa, Ontario, Canada; SickKids Inflammatory Bowel Disease Centre, Division of Gastroenterology, Hepatology, and Nutrition, The Hospital for Sick Children, Toronto, Ontario, Canada; Child Health Evaluative Sciences, SickKids Research Institute, The Hospital for Sick Children, Toronto, Ontario, Canada; Departments of Medicine and Community Health Sciences, University of Calgary, Calgary, Alberta, Canada; Department of Medicine, McMaster University, Hamilton, Ontario, Canada; Department of Gastroenterology and Hepatology, Temerty Faculty of Medicine, University of Toronto, Toronto, Ontario, Canada; SickKids Inflammatory Bowel Disease Centre, Division of Gastroenterology, Hepatology, and Nutrition, The Hospital for Sick Children, Toronto, Ontario, Canada; Child Health Evaluative Sciences, SickKids Research Institute, The Hospital for Sick Children, Toronto, Ontario, Canada; ICES, Toronto, Ontario, Canada; Department of Paediatrics, Temerty Faculty of Medicine, University of Toronto, Toronto, Ontario, Canada; Institute of Health Policy, Management, and Evaluation, Dalla Lana School of Public Health, University of Toronto, Toronto, Ontario, Canada; Department of Internal Medicine, Max Rady College of Medicine, Rady Faculty of Health Sciences, University of Manitoba, Winnipeg, Manitoba, Canada; IBD Clinical and Research Centre, University of Manitoba, Winnipeg, Manitoba, Canada; Division of Gastroenterology and Hepatology, McGill University Health Centre IBD Centre, McGill University, Montréal, Quebec, Canada; Departments of Medicine and Community Health Sciences, University of Calgary, Calgary, Alberta, Canada; Departments of Medicine, Clinical Health, and Epidemiology, Dalhousie University, Halifax, Nova Scotia, Canada; Departments of Medicine and Community Health Sciences, University of Calgary, Calgary, Alberta, Canada; Crohn’s and Colitis Canada, Toronto, Ontario, Canada; Division of Gastroenterology and Hepatology, Mount Sinai Hospital, University of Toronto, Toronto, Ontario, Canada; Department of Community Health and Epidemiology, University of Saskatchewan, Saskatoon, Saskatchewan, Canada; College of Nursing, University of Saskatchewan, Saskatoon, Saskatchewan, Canada; Crohn’s and Colitis Canada, Toronto, Ontario, Canada; Department of Neuroscience, McGill University, Montreal, Quebec, Canada; Department of Gastroenterology and Hepatology, Temerty Faculty of Medicine, University of Toronto, Toronto, Ontario, Canada; Department of Medicine, Temerty Faculty of Medicine, University of Toronto, Toronto, Ontario, Canada; Department of Gastroenterology and Clinical Nutrition, The Royal Children’s Hospital Melbourne, Parkville, Australia; SickKids Inflammatory Bowel Disease Centre, Division of Gastroenterology, Hepatology, and Nutrition, The Hospital for Sick Children, Toronto, Ontario, Canada; Child Health Evaluative Sciences, SickKids Research Institute, The Hospital for Sick Children, Toronto, Ontario, Canada; SickKids Inflammatory Bowel Disease Centre, Division of Gastroenterology, Hepatology, and Nutrition, The Hospital for Sick Children, Toronto, Ontario, Canada; Child Health Evaluative Sciences, SickKids Research Institute, The Hospital for Sick Children, Toronto, Ontario, Canada; SickKids Inflammatory Bowel Disease Centre, Division of Gastroenterology, Hepatology, and Nutrition, The Hospital for Sick Children, Toronto, Ontario, Canada; Child Health Evaluative Sciences, SickKids Research Institute, The Hospital for Sick Children, Toronto, Ontario, Canada; Departments of Medicine and Community Health Sciences, University of Calgary, Calgary, Alberta, Canada; Departments of Medicine and Community Health Sciences, University of Calgary, Calgary, Alberta, Canada; Crohn’s and Colitis Canada, Toronto, Ontario, Canada; Crohn’s and Colitis Canada, Toronto, Ontario, Canada; Crohn’s and Colitis Canada, Toronto, Ontario, Canada; Crohn’s and Colitis Canada, Toronto, Ontario, Canada; Crohn’s and Colitis Canada, Toronto, Ontario, Canada; Department of Internal Medicine, Max Rady College of Medicine, Rady Faculty of Health Sciences, University of Manitoba, Winnipeg, Manitoba, Canada; IBD Clinical and Research Centre, University of Manitoba, Winnipeg, Manitoba, Canada; Department of Community Health Sciences, Max Rady College of Medicine, Rady Faculty of Health Sciences, University of Manitoba, Winnipeg, Manitoba, Canada; CancerCare Manitoba Research Institute, Winnipeg, Manitoba, Canada

**Keywords:** Crohn’s Disease, Cancer, Screening, Surveillance, Ulcerative Colitis

## Abstract

Cancer is a major cause of morbidity and mortality among people with inflammatory bowel disease (IBD). Intestinal cancers may arise as a complication of IBD itself, while extra-intestinal cancers may arise due to some of the immunosuppressive therapies used to treat IBD. Colorectal cancer (CRC) and small bowel cancer risks remain elevated among persons with IBD as compared to age-and sex-matched members of the general population, and the lifetime risk of these cancers is strongly correlated to cumulative intestinal inflammatory burden. However, the cumulative risk of cancer, even among those with IBD is still low. Some studies suggest that IBD-CRC incidence has declined over the years, possibly owing to improved treatment standards and improved detection and management of early neoplastic lesions. Across studies of extra-intestinal cancers, there are generally higher incidences of melanoma, hepatobiliary cancer, and lung cancer and no higher incidences of breast cancer or prostate cancer, with equivocal risk of cervical cancer, among persons with IBD. While the relative risks of some extra-intestinal cancers are increased with treatment, the absolute risks of these cancers remain low and the decision to forego treatment in light of these risks should be carefully weighed against the increased risks of intestinal cancers and other disease-related complications with undertreated inflammatory disease. Quality improvement efforts should focus on optimized surveillance of cancers for which surveillance strategies exist (colorectal cancer, hepatobiliary cancer, cervical cancers, and skin cancers) and the development of cost-effective surveillance strategies for less common cancers associated with IBD.

**Key Points**

1. Colorectal cancer occurs 1.5–2 times more often in those with IBD than comparable members of the general population and is responsible for as much as 15% of IBD-related deaths.
2. Colorectal cancer risk is strongly associated with cumulative colorectal mucosal inflammatory burden encompassing IBD duration, extent of colorectal involvement, colitis severity, and frequency/chronicity of active disease.
3. Extra-intestinal cancers may complicate IBD treatment with chronic immunosuppressive therapies, due to reduced immune surveillance and/or a direct effect of the medications.
4. Thiopurines have been associated with increased risks of lymphoma and non-melanoma skin cancers. The risks of melanoma and cervical cancer in association with chronic immunosuppressive therapies remain uncertain.
5. Hepatobiliary cancer risk is significantly higher among those with IBD, particularly among persons with co-morbid primary sclerosing cholangitis. Emerging data also suggest a higher risk of lung cancer among those with IBD.
6. While the relative risks of certain cancers are increased in those with IBD, the absolute risks of those cancers remain low and should not be a deterrent to effective therapies to treat IBD.
7. Systematic cancer surveillance strategies currently exist for colorectal cancer, hepatobiliary cancers (if co-morbid primary sclerosing cholangitis), cervical cancers, and skin cancers. Cost-effective surveillance strategies do not currently exist for less common cancers, such as small bowel cancers.
8. Endoscopic surveillance and management of colorectal neoplasia have evolved considerably over the past 10–15 years, leading to higher rates of endoscopic resection of neoplastic lesions and lower rates of surgery. Surgery is now reserved for very high-risk neoplasia cases.
9. Post-colonoscopy colorectal cancers (those arising within three years of a colonoscopy) account for up to 50% of colorectal cancers in persons with IBD, which may be because of inadequate surveillance frequency, suboptimal colonoscopy quality, or altered tumour biology. Regular, high-quality surveillance colonoscopy is important to promote detection and management of neoplastic lesions and reduce the risk of missed cancers.
10. As people with IBD are living longer, gastroenterologists will experience a higher burden of individuals who develop cancer; this may impact treatment decisions/options. As the evidence accumulates, IBD care providers will need to revise their understanding of the implications of current IBD practice on cancer risk and accordingly modify preventive and treatment strategies across the spectrum of cancers observed in these individuals.

## INTRODUCTION

Intestinal and extra-intestinal cancers can arise as complications of inflammatory bowel disease (IBD) and its treatments, respectively. Cancer is one of the most common causes of death in this population (1,2), and cancer prevention is one of the major goals of IBD management. To-date, the primary focus has been on the prevention of colorectal cancer (CRC). This focus may largely relate to the historical preponderance of CRC as compared to other cancers in individuals with IBD, which may have resulted from the absence of highly effective treatments for IBD until the early 2000s. Over the past 20 years, multiple classes of effective biologic and targeted therapies that induce mucosal healing have been introduced (3–17), and there have been paradigm shifts in how we use these therapies (see Murthy et al. in this volume). Additionally, improved endoscopic techniques to detect and remove early neoplastic lesions have altered the relative potential for disease-related versus treatment-related cancers. The evidence for the effect of this shift on cancer-related morbidity and mortality may take decades to realize. As the evidence accumulates, gastroenterologists and other IBD care providers will need to revise their understanding of the implications of current IBD practice on cancer risk and accordingly modify preventive and treatment strategies across the spectrum of cancers observed in these individuals. This change will become increasingly important for shared decision-making between providers and persons with IBD. In the present article, we review the epidemiology of cancer in people with IBD, the impact of current treatments on cancer risk, and existing cancer preventive strategies in clinical practice.

## EPIDEMIOLOGY OF CANCER IN IBD

The risk of some, but not all, cancers are increased among individuals with IBD. The most consistent evidence demonstrates increased risks of: (i) CRC among those with colonic involvement, varying with the extent, severity, and duration of colonic inflammation; (ii) small bowel cancer among those with Crohn’s disease; (iii) lymphoma and non-melanoma skin cancer among those treated with thiopurine medications; and (iv) to some extent, melanoma among those treated with anti Tumor Necrosis Factor (anti-TNF) biologic therapy. Children diagnosed with IBD have also been reported to have increased cancer risk in their adult life.

No increase in cancer risk has been reported with vedolizumab or ustekinumab, or with tofacitinib so far among individuals with IBD, although more data are required as these therapies are relatively recent additions to the IBD armamentarium. In addition, no increased risk of cancer with anti-TNF therapy after an index cancer diagnosis has been demonstrated, suggesting lengthy post-cancer drug holidays are unlikely to be necessary. Importantly, the absolute risk of cancers is small even for the cancers demonstrated to be increased with therapy, which is important information for shared decision-making between providers and persons with IBD.

In the following sections, we outline important studies that have influenced our understanding of the epidemiology of cancer in IBD, focusing on population-based studies (when available), and highlighting important Canadian studies on this topic. A summary of ranges of estimates of risks across studies included in this article is listed in Table 1.

**Table 1.** Summary of recent studies comparing the risk of cancers in people with and without inflammatory bowel disease (IBD). When estimates from multiple studies are reported, data are presented as a range of effect estimates. When estimates come from a single study, they are reported as effect estimates with 95% confidence intervals (CI)

Cancer type	Diagnosis at any age	Pediatric-onset	Comments
Colorectal	**IBD,** IRRs: 1.1–1.8 (18–20) OR: 1.78 (1.57, 2.02) (21) HR: 1.95 (1.65, 2.30) (22) **CD,** IRRs: 1.6–1.7 (18,23) **UC,** IRRs: 1.6–1.7 (18,23)	**IBD,** IRR: 20.29 (17.20, 25.90) (24)	Most studies show statistically significant increased risk among persons with IBD.
Small intestine	**IBD,** IRR: 7.4 (5.6, 9.8) (20) OR: 6.6 (4.7, 9.4) (21) **CD,** HR: 19.7 (10.5, 36.7)	**IBD,** IRR: 16.2 (3.5, 74.7) (24)	Most studies show statistically significant increased risk among persons with IBD.
Lymphoma	**IBD,** IRRs: 1.4–2.0 (20) **CD,** IRR: 2.4 (1.8, 3.2) (25)	**IBD,** IRR: 3.1 (1.9, 5.1) (24)	Meta-analyses showed statistically significant increased risk among persons with IBD, but many individual population-based studies were varied.
Melanoma	**IBD,** IRRs: 1.2–1.3 (20,26) OR: 0.9 (0.8, 1.1) (21) HR: 1.0 (0.7, 1.5) (27) **CD,** IRRs 1.4–1.8 (28,29) ORs: 1.1–5.2 (28) **UC,** IRRs: 1.1–1.2 (28) OR: 0.9 (0.8, 1.1) (28)	**IBD,** IRR: 2.1 (1.3, 3.3) (24)	Studies show statistically significant increased risk among individuals with pediatric-onset IBD.
Non-melanoma skin cancer	**IBD,** IRRs: 1.5–1.6 (26,30) OR: 1.03 (0.97, 1.10) (21) **CD,** IRR: 2.3 (1.4, 3.8) (25) **UC,** IRR: 1.4 (1.1, 1.7) (25)	**IBD,** IRR: 3.6 (2.0, 6.7) (24)	Most studies have shown a modest association of IBD with melanoma.
Cervical	**IBD,** OR: 0.7 (0.6, 0.8) (21) **CD,** IRRs: 1.3–1.6 (20,25,31) **UC,** IRRs: 0.6–1.0 (20,25)		Inconsistent increased risk of cervical cancer.
Hepatobiliary	**IBD,** IRR: 2.5 (1.8, 3.5) (20) OR: 7.4 (5.6, 9.8) (21) **CD,** IRRs: 1.8–5.2 (32) **UC,** IRRs: 0.7–5.6 (32–34)	**IBD,** IRR: 55.5 (19.6, 157.0) (24)	Most studies show statistically significant increased risk among persons with IBD.
Lung	**IBD,** OR: 4.0 (3.5, 4.6) (21) **CD,** IRRs: 1.5–1.8 (20,25,33) **UC,** IRR: 0.4 (0.2, 0.7) (33)		Studies generally show statistically significant increased risk among persons with Crohn’s disease but not ulcerative colitis.
Breast	**IBD,** IRR: 1.0 (0.9, 1.2) (20) OR: 0.7 (0.6, 0.8) (21)		Studies generally show no association between IBD and breast cancer.
Prostate	**IBD,** IRR: 1.0 (0.9, 1.2) (20) OR: 0.6 (0.6, 0.7) (21)		Studies generally show no association between IBD and risk of prostate cancer.

Abbreviations: CD, Crohn’s disease; UC, ulcerative colitis.

### Intestinal Cancers

Intestinal cancers arise in people with IBD as a result of cumulative carcinogenic DNA damage from chronic or recurrent bouts of bowel inflammation (35–38). Due to the high rate of colonic involvement in IBD (100% in ulcerative colitis, 70% in Crohn’s disease), CRC accounts for more than 95% of gastrointestinal tract malignancies in IBD and up to 15% of IBD-related deaths (2,39). Recognized disease-specific risk factors for CRC in those with IBD include longer disease duration (18,40–44), more extensive colorectal involvement (18,45), more severe colorectal inflammation (46–49), pseudopolyps (47) and chronic mucosal scarring (50), all of which are markers of cumulative colorectal inflammatory burden, which is now thought to be the most important factor overall (51,52). Other important CRC risk factors in persons with IBD include personal history of colorectal neoplasia (53,54), family history of CRC in a first-degree relative (55,56), and co-morbid primary sclerosing cholangitis (57). A normal colonoscopy (no inflammatory or neoplastic changes), particularly consecutive normal colonoscopies, is associated with a reduced risk of future advanced CRC (50,58).

Most population-based studies have reported CRC risk to be elevated among persons with IBD as compared to age- and sex-similar persons without IBD (18,20,21,23,59). A meta-analysis of population-based studies up to 2009 reported a standardized incidence ratio (SIR) of 1.7 (95% confidence interval [CI]: 1.2, 2.2) for CRC among persons with IBD relative to the general population, which was similar across Crohn’s disease and ulcerative colitis (18). High-risk groups were individuals with extensive colitis (SIR: 6.4; 95% CI: 2.4, 17.5) and diagnosis with IBD <30 years old (SIR: 7.2; 95% CI: 2.9, 17.8). Cumulative risks of CRC in this study were 1% and 2% after 10 and 20 years, respectively.

Since then, multiple population-based studies of CRC risk in IBD have been reported. A study from Northern California conducted between 1998 and 2010, based on the Kaiser Permanente database, reported SIRs for CRC among persons with Crohn’s disease and ulcerative colitis of 1.6 (Crohn’s disease, 95% CI: 1.2, 2.0; ulcerative colitis, 95% CI: 1.3, 3.0) (23). Conversely, adjusting for age, sex and calendar time, a nationwide study from Denmark did not find an increased CRC risk in their IBD population as compared to non-IBD members from the general population (SIR: 1.07; 95% CI: 0.95, 1.21) and actually reported a lower CRC risk in their IBD population by 1999–2008 (SIR: 0.57; 95% CI: 0.41, 0.80) (42). Unique characteristics of their cohort, including a high propensity for early colectomy, may have influenced their findings. Recent population-based studies from Canada have reported similarly increased risk for CRC among persons with IBD, as compared to age- and sex-matched controls without IBD (Ontario 2017–2020, SIR: 1.78; 95% CI: 1.64, 1.95 (20); Alberta 2002–2018, OR: 1.78; 95% CI: 1.57, 2.02 (21); Manitoba 1987–2012 (cases also matched to controls by area of residence, and analysis adjusted for prior history of lower gastrointestinal endoscopy, frequency of healthcare visits, and socioeconomic status), adjusted hazard ratio (aHR): 1.95; 95% CI: 1.65, 2.30) (60). A meta-analysis of three population-based studies reported a pooled rate ratio (RR) of 20.29 for CRC (95% CI: 15.90, 25.90) among persons with pediatric-onset IBD as compared to similar persons without IBD; the higher relative risk in this study may relate to a low baseline CRC risk in a younger population as well as longer IBD duration in many individuals (24).

Several population-based studies have further reported a significant decline in CRC incidence over time among persons with IBD (20,42), although others have not (23,40,60). A meta-analysis of population-based studies conducted before 2009 reported a pooled CRC incidence of 2% by 20 years of disease (18), which is considerably lower than the earlier reported CRC incidence of 8% at 20 years across population-based and referral centre studies (44). A Danish national study reported a declining SIR for CRC in their IBD population between 1978 and 2008 (42). Conversely, a study from Northern California reported stable CRC incidence in their IBD cohort between 1998 and 2010 (23). A recent population-based study from Ontario reported that CRC incidence steadily declined between 1994 and 2020 among persons with ulcerative colitis (average annual percentage change [AAPC]: −1.95; 95% CI: −2.59, −1.30) but not among persons with Crohn’s disease (AAPC: −1.04; 95 % CI: −2.91, −0.87) (20). Notably, CRC incidence similarly declined among persons without IBD during this period, and the CRC RR in persons with IBD as compared to age- and sex-matched persons without IBD during the 2017–2020 period was similar to what has been reported in earlier studies, suggesting that factors unrelated to IBD treatment may have led to the observed decline.

CRC-related mortality has also been reported to be higher among persons with IBD across studies. The aHR for mortality among persons with IBD in a population-based study from Manitoba was 2.15 (95% CI: 1.60, 2.89) (60). Similarly, a study from Northern California between 1998 and 2008 reported standardized mortality ratios for CRC of 2.3 (95% CI: 1.6, 3.0) in individuals with Crohn’s disease and 2.0 (95% CI: 1.3, 2.7) among individuals with ulcerative colitis (23). A Danish medical registry study reported 5-year adjusted mortality RRs for persons with Crohn’s disease of 1.26 (95% CI: 1.07, 1.49) and ulcerative colitis of 1.14 (95% CI: 1.03, 1.27) (61). IBD was an independent risk factor for death after CRC diagnosis in studies from Japan (62) and Manitoba (63). CRC-related mortality was reported to have declined several-fold among persons with IBD between 1960 and 2004 in a population-based study from Sweden (40). Conversely, the study from Manitoba reported no decline in the aHR for CRC-related death between 1987–1993 and 2002–2012 periods in persons with IBD as compared to persons without IBD, matched by age, sex, and area of residence (60).

Small bowel carcinomas account for 1% to 5% of all gastrointestinal tract cancers in persons with Crohn’s disease and have been historically reported to occur at a 20–30-fold higher rate as compared to persons without IBD (64,65). A recent population-based study from Manitoba reported a hazard ratio (HR) for small bowel cancer of 19.7 (95% CI: 10.5, 36.7) among persons with Crohn’s disease, which was lower for those above 65 years of age (HR: 8.41; 95% CI: 3.05, 23.2) than among younger individuals (age <50, HR: 44.5; 95% CI: 9.88, 200; 50–64 HR: 30.3; 95% CI:10.0, 91.7), and highest for ileal adenocarcinoma (HR: 125.7; 95% CI: 16.6, 950.7). Estimates from Ontario (2017–2020) (20) and Alberta (2002–2018) (21) have reported RRs for small bowel cancer among persons with IBD of 7.39 (95% CI: 5.58, 9.79) and 6.59 (95% CI: 4.65, 9.35), respectively, as compared to matched controls. Among persons with pediatric-onset IBD, a meta-analysis of population-based studies reported a pooled RR of 16.20 (95% CI: 3.52, 74.66) for small bowel cancers. Importantly, the high relative rate for small bowel cancers may largely be due to the low baseline rate of small bowel cancers in the general population; similar to other cancers observed more frequently among persons with IBD, the absolute incidence of these cancers remains low.

### Summary of Intestinal Cancer Epidemiology

CRC incidence continues to be 1.5–2 times higher among persons with IBD as compared to age- and sex-matched members of the general population; a risk ratio that has not appreciably changed over time. This ratio suggests that the observed downward trend in absolute CRC incidence in persons with IBD over the past three decades in several studies may be more related to changing genetic and/or environmental risk factors as opposed to changes in IBD treatment. Increased uptake of colonoscopy screening in society may have played a role in declining CRC risk over time. CRC-related mortality also remains roughly two times higher among persons with IBD as compared to persons without IBD who develop CRC suggesting either more aggressive tumour biology; a higher rate of advanced, incurable cancers at diagnosis among persons with IBD; or a direct effect of increased CRC incidence. Persons with IBD remain at substantially higher risk of developing small bowel cancer as compared to their non-IBD counterparts, likely due to greater difficulty in managing small bowel inflammation and the absence of screening strategies for detecting early small bowel neoplasms. However, the absolute risk of small bowel cancer remains exceedingly low; overall, intestinal cancer risk is likely to always be dominated by CRC risk. Strategies aimed at reducing the CRC potential of IBD, and at improving identification of individuals at higher risk of developing CRC to prioritize colonoscopy or surgery, will be required to attenuate the difference in CRC risk between persons with and without IBD.

### Extra-Intestinal Cancers

Extra-intestinal cancers in persons with IBD may arise as a result of reduced immune surveillance in the setting of chronic immunosuppressive therapy or as a direct effect of such therapies. Thiopurines, for example, are photosensitizers and lead to development of mutagenic reactive oxygen species (66). The most commonly reported extra-intestinal cancers in those with IBD are lymphoma, melanoma, non-melanoma skin cancers (NMSC), cervical cancer, and hepatobiliary cancers (67). In a recent meta-analysis of 15 studies, the overall risk of extra-intestinal cancers was found to be increased in persons with Crohn’s disease (incidence rate ratio [IRR]: 1.43; 95% CI: 1.26, 1.63) and ulcerative colitis (IRR: 1.15; 95% CI: 1.02,1.31) (25).

### Lymphoma

Many population-based studies have not shown an overall association between IBD and lymphoma risk (68–70). However, a recent meta-analysis of 15 population-based studies reported an increased risk of hematologic malignancies among persons with Crohn’s disease (IRR, 2.40; 95% CI: 1.81, 3.18). A meta-analysis of three population-based studies further reported an increased risk for lymphoid cancer among persons with pediatric-onset IBD (pooled RR: 3.10; 95% CI: 1.88, 5.10) (24).

Thiopurine use (azathioprine and 6-mercaptopurine) in persons with IBD has been consistently associated with an increased risk of lymphoma (71–73). In a recent meta-analysis of studies comparing persons with IBD receiving thiopurines to members of the general population, the SIR for lymphoma was 4.92 (95% CI: 3.10, 7.78), ranging from 2.80 (95% CI: 1.82, 4.32) in eight population studies to 9.24 (95% CI: 4.69, 18.2) in 10 referral studies (71). Population-based studies demonstrated an increased risk among current thiopurine users (SIR: 5.71; 95% CI: 3.72, 10.1) but no increased risk among former users (SIR: 1.42; 95% CI: 0.86, 2.34). The magnitude of risk only became significant after one year of cumulative exposure. Persons younger than 30 years of age had the highest relative risk (SIR: 6.99; 95% CI: 2.99, 16.4), whereas the absolute risk was highest in persons older than 50 years (28.24 cases per 10,000 PY, with a relative risk of 4.78). Males carried a greater lymphoma risk than females (males, SIR: 4.50; 95% CI: 3.71, 5.40; females, SIR: 2.29; 95% CI: 1.69, 3.05).

A more recent French nationwide study of 189,289 adults with IBD followed for a median of 6.7 years reported a 2–3-fold higher risk of lymphoma in association with thiopurine (aHR: 2.60; 95% CI: 1.96, 3.44) and anti-TNF (aHR: 2.41; 95% CI: 1.60, 3.64) monotherapy, and a synergistic effect with combination therapy (aHR: 6.11; 95% CI: 3.46, 10.8). The aHR among those receiving combination therapy was 2.35 (95% CI: 1.31, 4.22) compared to thiopurine monotherapy and 2.53 (aHR: 2.53; 95% CI: 1.35, 4.77) compared to anti-TNF monotherapy (74). Notably, other studies have not found an independent association between anti-TNF therapy and lymphoma risk (75–77). To-date, there is no data supporting an increased lymphoma risk with other classes of biologic therapy.

Across Ontario (2017–2020), the SIR for non-Hodgkin’s lymphoma (NHL) and Hodgkin’s lymphoma among persons with IBD, relative to age- and sex-matched controls, was found to be 1.43 (95% CI: 1.23, 1.65) and 1.95 (95% CI: 1.00, 3.81), respectively (20). For NHL, this risk increased over a 25-year period (AAPC: 2.26; 95% CI: 0.924, 3.62), whereas it remained unchanged in the general population (AAPC: −0.071; 95% CI: −0.486, 0.346). Across Alberta (2002–2018), the OR for hematologic malignancies was 1.37 (95% CI: 1.21, 1.56) for persons with IBD relative to age- and sex-matched controls (21). To what extent the higher rates of lymphoma in these populations were driven by immunosuppressive therapies is being actively investigated.

Furthermore, research with the DEVELOP registry has demonstrated a clear association between thiopurine use (but not biologic monotherapy) and lymphoma risk in children with IBD (78). While most thiopurine-related lymphomas are Epstein-Barr virus-associated B-cell lymphomas (72), young males have also been reported to develop fatal early post-mononucleosis B-cell lymphomas (if they are seronegative for Epstein-Barr virus prior to initiating thiopurine therapy) (72) and hepatosplenic T-cell lymphomas (γδ variant) following exposure to thiopurines (79).

### Melanoma

Multiple studies have demonstrated a modest association of IBD with melanoma, although the impact of medical therapies on melanoma risk remains uncertain. A meta-analysis of 12 cohort studies between 1940 and 2009, comprising 172,837 persons with IBD, reported an increased risk of melanoma among persons with Crohn’s disease (seven studies, IRR: 1.80; 95% CI: 1.17, 2.75) and ulcerative colitis (seven studies, IRR: 1.23; 95% CI: 1.01, 1.50) (28). The pooled incidence rate of melanoma was 27.5 per 100,000 PY (95% CI: 19.9, 37.0). A meta-analysis of four population-based cohorts reported a pooled IRR of 1.14 (95% CI: 0.92, 1.42) among persons with ulcerative colitis and 1.52 (95% C: 1.03, 2.23) among persons with Crohn’s disease (25). A meta-analysis of three population-based studies among persons with pediatric-onset IBD reported a pooled RR of melanoma of 2.05 (95% CI: 1.27, 3.29) (24).

A U.S. administrative claims database study comparing 108,579 people with IBD to matched controls without IBD between 1997–2009, similarly reported an IRR of 1.29 for melanoma among those with IBD (95% CI: 1.09, 1.53) (26). A nationwide Danish cohort registry study further reported an increased risk of melanoma among 13,756 people with Crohn’s disease between 1978 and 2010 (SIR: 1.4; 95% CI: 1.0, 1.9) (29). Notably, more recent Canadian population-based studies have not reported an increased risk of melanoma among persons with IBD as compared to age- and sex-matched controls (Ontario [2017–2020], SIR: 1.23; 95% CI: 0.857, 1.77 (20); Alberta [2008–2018], OR: 0.89; 95% CI: 0.75, 1.07 (21); Manitoba [1986–2018], HR: 1.04; 95% CI: 0.71, 1.53 (27)).

Most studies have not supported an increased risk of melanoma in association with thiopurine therapy (26,80,81). A meta-analysis of 13 studies in 149,198 persons with IBD reported a non-significant RR of 1.22 (95% CI: 0.90, 1.65) for melanoma in association with thiopurine use. However, several studies have demonstrated a slightly higher risk of melanoma in association with anti-TNF biologic therapy (26,27,82). A nested case–control study of a large IBD cohort based on U.S. claims data reported an increased odds of melanoma in users of biologic therapy of 1.88 (95% CI: 1.08, 3.29). However, a meta-analysis of eight cohort studies among 51,231 persons with IBD did not find a significant association between anti-TNF therapies and melanoma risk (83). A population-based study from Quebec also did not find an association between anti-TNF therapy and melanoma risk (84).

### Non-Melanoma Skin Cancer

NMSC, mainly basal cell carcinoma (BCC) and squamous cell carcinoma (SCC), are typically non-fatal cancers that are strongly linked to sun exposure. Studies using U.S. administrative health insurance claims data have reported a higher incidence of NMSC among persons with IBD compared with matched controls (IRR: 1.64; 95% CI: 1.51, 1.78 (85); IRR: 1.46; 95% CI: 1.40, 1.53 (26)). A recent population-based study from Manitoba also reported that the risks of BCC (HR: 1.53; 95% CI: 1.37, 1.70) and SCC (HR: 1.61; 95% CI: 1.29, 2.01) were significantly increased in persons with IBD, except for SCC in ulcerative colitis (27). However, this study also reported an increased risk of BCC predating ulcerative colitis diagnosis (OR: 1.32; 95% CI: 1.08, 1.60). A meta-analysis of 15 population-based studies reported IRR of 1.36 (95% CI: 1.05, 1.71) and 2.28 (95% CI: 1.36, 3.81) in persons with ulcerative colitis and Crohn’s disease, respectively (25). A meta-analysis of three population-based studies in persons with pediatric-onset IBD reported a pooled RR of NMSC of 3.62 (95% CI: 1.97, 6.66) (24).

Most studies have reported an increased risk of NMSC in association with thiopurine therapies (26,27,81,84,85). A meta-analysis of 13 studies comprising 149,198 individuals with IBD reported an excess risk of NMSC associated with thiopurine use (RR: 1.88; 95% CI: 1.48, 2.38). Deaths due to NMSC predominantly occur among individuals who continue thiopurines after NMSC diagnosis (86). The risk of NMSC with anti-TNF therapies remains unclear: Some studies have reported a marginally increased risk (27,82,85), while other studies have not (26,84).

### Cervical Cancer

Studies describing the risk of cervical neoplasia in people with IBD have shown conflicting results, with some reporting increased risk (87–91), some reporting lower risk (21,59, 92–94), and others reporting no association (20,95). A Danish nationwide cohort study of women diagnosed with ulcerative colitis (*n* = 18,691) or Crohn’s disease (*n* = 8,717) between 1979 and 2011, matched to 1,508,334 women from the general population, reported that women with ulcerative colitis had increased risks of low-grade (IRR: 1.15; 95% CI: 1.00, 1.32) and high-grade (IRR: 1.12; 95% CI: 1.01, 1.25) squamous intraepithelial lesions, and women with Crohn’s disease had increased risks of low-grade SIL (IRR: 1.26; 95% CI: 1.07, 1.48), high-grade SIL (IRR: 1.28; 95% CI: 1.13, 1.45) and cervical cancer (IRR: 1.53; 95% CI: 1.04, 2.27), compared with controls (91). However, a meta-analysis of three population-based studies reported no higher risk of cervical cancer among women with ulcerative colitis (IRR: 0.95; 95% CI: 0.60, 1.51) or Crohn’s disease (IRR: 1.58; 95% CI: 0.90, 2.77) (25). Notably, most studies have not adjusted for differences in cervical cancer screening rates.

A population-based study from Ontario (2017–2020) reported no increased risk of cervical cancer among persons with Crohn’s disease (SIR: 1.25; 95% CI: 0.829, 1.87) and a decreased risk of cervical cancer in persons with ulcerative colitis (SIR: 0.62; 95% CI: 0.38, 0.99) (20). A population-based study from Alberta (2002–2018) showed a decreased odds of cervical cancer among those with IBD (OR: 0.68; 95% CI: 0.61, 0.77) (21). An earlier population-based study from Manitoba reported no association between cervical abnormalities and ulcerative colitis (OR: 1.03; 95% CI: 0.77, 1.38), and only showed an increase in risk in women with Crohn’s disease if they were exposed to 10 or more prescriptions of oral contraceptives (OR: 1.66; 95% CI: 1.08, 2.54); this study adjusted for cervical cancer screening in the preceding five years (95).

In the Manitoba study, combined exposure to corticosteroids and immunosuppressants was associated with increased risk of cervical abnormalities (OR: 1.41; 95% CI: 1.09, 1.81) (95). A meta-analysis of eight studies comprising 77,116 individuals with IBD receiving immunosuppression further reported a 1.3-fold higher risk of cervical high-grade dysplasia among women with IBD as compared to healthy controls (OR: 1.34; 95% CI: 1.23, 1.46) (96). Furthermore, a Danish cohort study of 341,758 individuals with autoimmune diseases reported an increased risk of cervical cancer among those who had received a high cumulative dose of azathioprine (HR: 2.2; 95% CI: 1.2, 3.9) (97). The association between biologics and cervical neoplasia in people with IBD has not been extensively studied. One small study did not observe any cervical cancer cases among people exposed to anti-TNF therapy (94). Further research is required in this area.

### Hepatobiliary Cancers

Hepatobiliary cancers in people with IBD are more likely to occur as a complication of co-morbid liver or biliary disease—particularly primary sclerosing cholangitis—than as a complication of IBD itself or its treatments. A population-based study from Manitoba reported a higher incidence of hepatobiliary cancers in both Crohn’s disease (IRR: 5.22; 95% CI: 0.96, 28.5) and ulcerative colitis (IRR: 3.96; 95% CI: 1.05, 14.9) (59). However, a subsequent meta-analysis of eight population-based cohort studies comprising 17,052 individuals with IBD reported an increased risk of hepatobiliary cancers only among persons with ulcerative colitis (SIR: 2.58; 95% CI: 1.58, 4.22) (33). A Swedish national study also reported strong associations between bile duct cancers and ulcerative colitis (SIR: 5.6; 95% CI: 3.6, 8.4) (34).

A more recent meta-analysis of population-based studies reported pooled RRs of close to 3-fold for biliary tract cancers across three studies (ulcerative colitis, IRR: 2.93; 95% CI: 1.73, 4.96; Crohn’s disease, IRR: 2.93; 95% CI: 1.16, 7.41), but no increased risk of liver cancer across six studies (ulcerative colitis, IRR: 1.41; 95% CI: 0.99, 2.01; Crohn’s disease, IRR: 1.81; 95% CI: 0.88, 3.75). Strikingly, a meta-analysis of three population-based cohort studies in persons with pediatric-onset IBD reported a pooled RR of 55.45 (95% CI: 19.59, 156.99) for biliary tract cancers. Again, this heightened risk may largely relate to a lower baseline risk of biliary tract cancers in a younger population.

A recent population-based study from Ontario (2017–2020) reported a 2.5-fold increased risk of hepatobiliary cancers (SIR: 2.53; 95% CI: 1.84, 3.47) (20), which was similar in Crohn’s disease and ulcerative colitis, while a population-based study from Alberta reported a 7.4-fold increased risk (OR: 7.41; 95% CI: 5.58, 9.84) (21). There are no data on the risk of hepatobiliary cancers with biologic or other therapies in IBD.

### Other Cancers

An earlier meta-analysis of population-based studies reported a higher incidence of lung cancer (SIR: 1.82; 95% CI: 1.18, 2.81) and bladder cancer (SIR: 2.03; 95% CI: 1.14, 3.63) among people with Crohn’s disease, and a lower incidence of lung cancer (SIR: 0.39; 95% CI: 0.20, 0.74) among people with ulcerative colitis—as compared to persons without IBD (33). A more recent meta-analysis of 15 population-based studies reported a slightly higher risk of lung cancer in persons with Crohn’s disease (IRR: 1.53; 95 % CI: 1.23, 1.91), but otherwise no increased risks of lung cancer in persons with ulcerative colitis or of breast, prostate, or urogenital cancers in persons with Crohn’s disease or ulcerative colitis.

In a recent population-based study from Ontario, a weak association was observed between Crohn’s disease and lung cancer (SIR: 1.64; 95% CI: 1.37, 1.96), but no association was observed between IBD and either breast cancer (SIR: 1.04; 95% CI: 0.93, 1.15) or prostate cancer (SIR: 1.02; 95% CI: 0.88, 1.19) (20). Across Alberta, people with IBD showed a 4-fold higher odds of lung cancer (OR: 4.01; 95% CI: 3.46, 4.64), but slightly lower odds of breast cancer (OR: 0.72; 95% CI: 0.64, 0.81) and prostate cancer (OR: 0.64; 95% CI; 0.57, 0.73), relative to age- and-sex-matched controls (21). They also reported substantially higher odds of pancreas (OR: 7.79; 95% CI: 5.53, 10.97), kidney (OR: 2.05; 95% CI: 1.66, 2.52), and neurologic (OR: 4.58; 95% CI: 2.97, 7.04) cancers, but lower odds of bladder (OR: 0.68; 95% CI: 0.54, 0.87) and endometrial (OR: 0.48; 95% CI: 0.34, 0.66) cancers.

### Summary of Extra-Intestinal Cancer Epidemiology

Overall, the risks of extra-intestinal cancers appear to be higher among persons with IBD as compared to persons without IBD, which may be partly driven by chronic systemic inflammation and/or by the immunosuppressive therapies used to treat IBD. Whether individuals with IBD also have increased genetic susceptibility to these cancers remains unknown. Clear signals for increased cancer risks among persons with IBD exist for lymphoma and NMSC (primarily in association with thiopurine therapy), hepatobiliary cancers (mainly in association with co-morbid primary sclerosing cholangitis), and lung cancer. However, the risks of other extra-intestinal cancers have been variable across studies and require further elucidation. A multi-provincial Canadian population-based study aims to investigate the association between anti-TNF and thiopurine therapies and cancer risks in the IBD population. Of particular concern is the potential risk of fatal lymphomas in association with thiopurine therapies in young males. Until more data are available, practitioners should limit excess thiopurine exposure for people with IBD, particularly the elderly and young males, and to maintain vigilance in minimizing the risk of extra-intestinal cancers, such as through following appropriate screening measures and monitoring for early signs of cancer occurrence.

## CANCER PREVENTION IN IBD

### Screening for CRC

Prevention of CRC and CRC-related death have long been important goals in the management of IBD. Historical reports of widespread DNA damage in chronically inflamed colorectal mucosa (i.e., field cancerization) (35–37), a propensity towards irregular and indistinct growth patterns of colorectal neoplasia that could evade detection during colonoscopy (98–100), and high rates of missed CRC during colonoscopy (101–110), in conjunction with epidemiologic evidence of increased CRC incidence above the background risk beyond the first decade of disease among people with colonic IBD (18,43,44), have led to societal recommendations for lifelong intensive colonoscopy surveillance in persons with IBD affecting the colorectum, beginning at 8–10 years following disease diagnosis (111–115). Surveillance intervals of 1–5 years are now recommended for most people with colorectal IBD, guided by established CRC risk factors (116). A detailed summary of colonoscopy surveillance recommendations across multiple societies is provided in a review by Shah and Itzkowitz (117). While there is limited direct evidence of the utility of colonoscopy surveillance, the observed reduction in CRC incidence among people with IBD over time in some studies could, at least partly, be attributed to improved surveillance standards, with increased recognition and treatment of early colorectal neoplasia.

Updated guidance favours endoscopic management of all colorectal neoplasia with clearly delineated borders and without advanced features suggestive of invasive cancer or submucosal fibrosis, as well as continued endoscopic surveillance, as opposed to surgery, for individuals without high-risk neoplastic findings (multi-focal invisible dysplasia, invisible high-grade dysplasia, unresectable colorectal neoplasia) or difficult to surveille colons (116,118). Referral to an advanced endoscopist or a specialized centre for advanced endoscopic approaches is recommended for individuals with poorly delineated, invisible, or difficult-to-resect lesions prior to considering definitive surgery (116). Dye-spray chromoendoscopy and virtual chromoendoscopy techniques are being increasingly advocated as primary screening and surveillance adjuncts to high-definition colonoscopy (116,119,120). While taking extensive non-targeted biopsies has been an adjunct to colonoscopy surveillance in IBD for many years, its utility for routine screening and surveillance is being increasingly questioned due to its low yield for detecting neoplasia and increasing evidence that most colorectal neoplasias are visible with modern endoscope technology (116,118,121).

Importantly, several studies have demonstrated high rates of post-colonoscopy CRC (CRC diagnosed within three years following a colonoscopy) among persons with IBD, ranging between 25% and 50% of all CRC diagnosed in this population across studies (63,122–124). To what extent these findings are due to altered biology of CRC in IBD versus missed colorectal neoplasms during colonoscopy is unknown. These findings highlight the importance of careful inspection during colonoscopy under optimized conditions for neoplasia detection and of appropriate management of identified neoplastic lesions by specialists who are trained to do so (116). It further highlights the importance of intensive colonoscopy surveillance in those with higher risk, and the need for non-invasive tests to detect CRC and CRC precursors in people with IBD.

There are inconsistent data regarding the chemopreventive benefits of IBD therapies, including 5-aminosalicylate acids therapies (125–127) and thiopurines (128–131). Thus, no firm recommendations exist for the use of these agents solely for the purposes of CRC prevention.

### Screening for Small Bowel Cancer

There are no screening or surveillance recommendations for small bowel cancers in IBD due to the lower incidence as compared to CRC and perceived cost-ineffectiveness of routine screening (2,64). Fortunately, most small bowel cancer in Crohn’s disease occurs in the distal terminal ileum, which should be routinely inspected during colonoscopy performed for any reason in those with Crohn’s disease, offering some opportunity for small bowel surveillance (132).

### Screening for Hepatobiliary Cancer

The American College of Gastroenterology (ACG) recommends screening for biliary tract and gall bladder cancers in the setting of co-morbid primary sclerosing cholangitis and IBD with ultrasound or magnetic resonance cholangiopancreatography along with CA19-9 measurement every six months (133). Prophylactic cholecystectomy is further recommended for gallbladder polyps larger than eight mm to prevent the development of gallbladder cancer (133). The impact of these strategies on hepatobiliary cancer-associated mortality remains unknown.

### Screening for Cervical Cancer

While it is unclear whether IBD or its treatments are truly associated with an increased risk of cervical cancer, the American College of Obstetricians and Gynecologists (134), ACG (135), and an expert panel (136) have recommended annual cervical cancer screening with Papanicolaou (Pap) tests for females with IBD receiving chronic immunosuppressive therapy. At a minimum, females with IBD should remain up to date with cervical cancer screening recommended for females in the general population. Traditionally rates of Pap testing have been lower among females with IBD on immunosuppressants (137). Being vaccinated against HPV (human papilloma virus) is recommended as per general population guidelines (135).

### Screening for Skin Cancer

The ACG recommends that all persons with IBD undergo regular skin surveillance for NMSC if they are receiving thiopurine therapy, particularly if they are over the age of 50, as well as regular skin surveillance for melanoma, irrespective of receipt of immunosuppressive therapy (135). They further recommend that all individuals initiating or already taking chronic immunosuppressive therapy to be evaluated by a dermatologist, to tailor further evaluations based on individual risk, in addition to performing regular self-examinations, and also use sunscreen that is protective against UVA and UVB (ultraviolet A and B) light in addition to wearing sun protective clothing (135).

### Summary of Cancer Screening Recommendations in IBD

At present, cancer screening recommendations in persons with IBD are best established for CRC and for biliary cancers in persons with co-morbid CRC, whereas guidance for other cancer screening is limited. It is reasonable for individuals with IBD, particularly those receiving chronic immunosuppressive therapy, to undergo regular skin exams and Pap testing with their family physician. The role of routine screening for small bowel cancers and lung cancers, as well as for other internal cancers, must weigh the benefits of screening against the risks and costs and should be individualized. Formal screening recommendations for these and other rare cancers in persons with IBD need to be developed. A summary of existing screening recommendations for IBD-relevant cancers is provided in Table 2.

**Table 2.** Summary of available surveillance strategies for intestinal and extra-intestinal cancers in persons with IBD

Cancer type	Surveillance method	Surveillance frequency
Colorectal	Colonoscopy	1–5 years, based on risk factors (116,117)
Skin Cancers	Skin exam	Annual if receiving thiopurine therapy (135)
Hepatobiliary	Ultrasound or MRCP, along with CA19-9 measurement	Every six months (133)
Cervical	Papanicolaou test	Annual if receiving immunosuppressive therapy; otherwise maintain up-to-date with screening (134–136)

## BIOLOGIC/IMMUNOMODULATOR USE AFTER MALIGNANCY DIAGNOSIS

Data are limited on the use of immunosuppressive therapies after a cancer diagnosis. Immunomodulators are currently not recommended within 2–5 years after a cancer diagnosis (138). Mounting evidence from large retrospective studies shows no increased risk of relapse (or new cancer) with anti-TNF therapies or thiopurines (138–142), nor with vedolizumab or ustekinumab (143–145). Ongoing use of biologics and/or immunomodulators after malignancy should be individualized based on the type of cancer and type of immunosuppression and should be a shared decision in consultation with the treating oncologist and/or surgeon.

## KNOWLEDGE GAPS AND FUTURE RESEARCH DIRECTIONS

1. Research in large cohorts with long-term follow-up is required to better understand cancer risks faced by those with IBD, particularly in relation to different classes of existing and emerging immunosuppressive therapies. Individual risk factors for treatment-related cancers need to be elucidated. Additionally, cancer risk from IBD itself needs to be distinguished from that related to IBD treatments.
2. A clearer understanding of the importance and optimal methods of cancer screening and prevention is needed. Better defined and more widely available screening and surveillance methods are required for extra-intestinal cancers. Cost-effective screening and surveillance methods are also needed for cancers that are not as easily identifiable in their early stages.
3. Better risk stratification tools to optimize colonoscopy delivery are also required to improve colorectal neoplasia detection in high-risk individuals and defer unnecessary colonoscopy in low-risk individuals.
4. A better understanding of the reasons underlying the high rate of post-colonoscopy colorectal cancers and methods to reduce this occurrence is required.

## PATIENT AND CAREGIVER PARTNER PERSPECTIVE

While the risk of developing cancer in persons living with IBD is small, there is still an increased risk of certain cancers that individuals with IBD need to be aware of. The risk of cancer can be associated with the disease itself or the medications used to treat IBD. Even though the prospect of being at a greater risk for cancer development can be worrying for some, patient partners welcome conversations with multidisciplinary providers regarding risk factors and recommended screening for cancers. Receiving this information can facilitate self-advocacy and informed, shared decision making about treatment options and disease management. Patient partners stressed the importance of cancer screening and surveillance to decrease the risk of cancer development or discovering the cancer in its early stages.

## POLICY IMPLICATIONS AND KEY ADVOCACY OUTCOMES

1. Individuals with IBD receiving immunosuppressive therapies (especially thiopurines and anti-TNF therapies) should receive regular screening and surveillance for potential treatment-related cancers where screening protocols exist.
2. Lifelong excellent control of bowel inflammation is important to reduce colorectal cancer risk; physicians should have access to the best treatment to control inflammatory burden as required.
3. Beyond treating the underlying IBD, careful high-quality colonoscopy examinations remain the best strategy for CRC prevention and should be available to all individuals with colorectal IBD.
4. Research should be encouraged and funded to develop non-invasive markers of neoplasia in IBD.
5. Shared decision-making should inform treatment choices and cancer risks for IBD, weighing the risks of untreated inflammatory disease against the small, but potentially serious, risks of extra-intestinal cancers with immunosuppressive therapies. The difference between the absolute risks and relative risks of various cancers should be highlighted during these discussions.
6. For individuals with IBD who develop cancer, regular communication between gastroenterologists and oncologists should be encouraged to optimize management.

## SUPPLEMENT SPONSORSHIP

This article appears as part of the supplement “The Impact of Inflammatory Bowel Disease in Canada in 2023”, sponsored by Crohn’s and Colitis Canada, and supported by Canadian Institutes of Health Research Project Scheme Operating Grant (Reference number PJT-162393).

## Data Availability

No new data were generated or analyzed in support of this review.
